# Understanding the Magnetic Exchange Pathways of Transition-Metal-Doped Monolayer TiS_2_ Using First-Principles Calculations

**DOI:** 10.3390/nano15181435

**Published:** 2025-09-18

**Authors:** P. J. Keeney, P. M. Coelho, J. T. Haraldsen

**Affiliations:** Department of Physics, University of North Florida, Jacksonville, FL 32224, USA; n01445026@unf.edu (P.J.K.); paulamariel.coelho@unf.edu (P.M.C.)

**Keywords:** density functional theory, magnetism, transition-metal dichalogenides, first-principles, Heisenberg model, RKKY interaction

## Abstract

The ideal crystal symmetry of the 1T-TiS_2_ lattice results in a non-magnetic structure. However, recent studies have demonstrated that it may become magnetic upon substitution with transition-metal (TM) atoms. In this study, we examine the mechanisms and interactions that allow magnetic exchange through the TiS_2_ matrix. Using density functional theory, we model the substitutional TM-doped TiS_2_ (TM = V, Cr, or Mn) system with varying spatial distances to examine the effects on the magnetic exchange. Since pristine 1T-TiS_2_ is weakly semiconducting, there is a possibility that the introduction of metallic atoms may induce an RKKY-like interaction. We find that the substitution of vanadium produces a standard exchange through the orbital interactions. However, the introduction of chromium and manganese may generate RKKY interactions with the conduction electrons. Overall, a more comprehensive understanding of how different dopants affect magnetic behavior and communicate through the lattice can enable the design of spintronic devices, which offer the potential for more energy-efficient technologies and a deeper understanding of low-dimensional systems.

## 1. Introduction

Advancements in technologies like machine learning, artificial intelligence, and cryptocurrency continue to increase energy demands [1,2,3], creating a need for further optimizations in energy transfer and memory storage density. The area of spintronics offers a pathway towards more efficient energy technologies by exploring the potential of utilizing low-energy spin interactions over electronic excitations [4,5,6]. For example, magnetic random access memory (MRAM) is a spintronic device that uses electron spin as the key mechanism for data storage [7,8,9]. While various hurdles must be overcome for spintronics and MRAM to replace traditional semiconductor-based memory, there is evidence that supports the idea that MRAM could be used to minimize the downsides of modern memory storage, such as energy loss and heat dissipation [10].

Developing successful spintronic devices requires materials that meet ideal specifications that range from an exact thickness of material to a precise magnetic moment to ground state magnetic behavior (ferromagnetic, antiferromagnetic, etc.) [4,5,6]. Therefore, 2D materials provide the perfect construct for versatility and design [11,12,13]. Further research on 2D materials could provide the precise specifications necessary for spintronic devices to achieve a memory density beyond the current state of 1G RAM, reaching a more competitive [14] or industry-standard number [11,12,13].

Such 2D materials enable the optimization of current systems, such as traditional flash memory and semiconductor memory, in addition to providing integral contributions toward innovative solutions (such as spintronics, valleytronics, etc.) [13,15,16,17]. Moreover, 2D materials offer more than reduced dimensionality. They are powerfully tunable and can give specific properties [18] that depend on various factors, such as exact stoichiometry of elements within a sample or defect engineering [19,20,21,22], external doping either during synthesis or post-synthesis [23,24,25], and even interactions between heterostructure interfaces [26,27]. Whether it is an exact bandgap required for an optoelectronic device or a specific magnetic ground state and/or moment to be incorporated within an SRAM device, 2D materials enable devices to be more optimized than ever.

Transition metal dichalcogenides (TMDs) are a family of van der Waals (vdW) materials characterized by their chemical formula of MX_2_, with M representing a transition metal (Ti, Mo, and W, etc.) and X representing a chalcogen (S, Se, Te) [28]. TMDs exhibit layers of chalcogen atoms between singular layers of transition metal atoms and often exist in a 1T (space group 164, $P\bar{3}m1$) or 2H (space group 194, $P6_{3}/mmc$) configuration [29].

TiS_2_ is a TMD that has been shown computationally to exhibit a controllable magnetic state through doping [30]. While TiS_2_ in its bulk form has been widely studied [25,31], questions remain regarding how doping will impact a TiS_2_ thin film or monolayer [32,33,34]. Among the various possible applications of TiS_2_ are uses within the field of spintronics, in ion batteries [35,36], and in photovoltaic devices [37,38,39]. Recent computational studies demonstrate how magnetism can be introduced into the TiS_2_ matrix via substitutional transition-metal doping [30,32,33,40]. Therefore, our goal is to expand on the existing literature and gain an understanding of how magnetism communicates through the TiS_2_ lattice.

In this study, we examine the nature of the magnetic exchange interactions within monolayer 1T-TiS_2_. To achieve this, we specifically investigate the effects of substituting transition-metal atoms into the Ti sublattice. Under ideal conditions, Ti is typically non-magnetic due to the standard 4+ oxidation state. However, if the Ti site is directly substituted with transition-metal (TM) atoms (V, Cr, Mn), the additional electrons dope the site and provide a net magnetism in the lattice. Using density functional theory, we model the exchange interaction of TiS_2_ with one and two TM atoms (TM = V, Cr, or Mn). In the two-TM-atom system, we examine the magnetic ground state and exchange interaction with varying spatial separations along the zig-zag direction. The goal is to determine how the electrons in the TiS_2_ lattice mediate the magnetic interactions. Since pristine TiS_2_ is weakly semiconducting, there is a possibility that the introduction of metallic atoms may introduce an RKKY-like interaction through the conduction electrons. In general, we find that the substitution of vanadium produces a standard exchange through the orbital interactions. However, the introduction of chromium and manganese may generate the presence of RKKY interactions with the conduction electrons, although only Cr produces a weakly metallic state.

## 2. Methodology

Density functional theory calculations performed within this paper are done with the QuantumATK software [41,42,43]. Each calculation employs a spin-polarized meta-generalized gradient approximation (SMGGA) and SCAN functional, utilizing a linear combination of atomic orbitals (LCAO) basis set. Compared to LDA and GGA, SCAN improves predictions of ground-state structures, magnetic moments, and electronic properties, especially in strongly correlated systems [44,45], and often matches or exceeds the accuracy of hybrid functionals but at a computational cost closer to GGA functionals [45]. A PseudoDojo pseudopotential is applied [43] as well as a *k*-point sampling of 3 × 3 × 1. The iteration control parameter is set to 10^−7^ Hartrees, and the maximum force for geometry optimization is 0.05 eV/Å. The benchmarking process ensures agreement with previously established experimental data for the pristine TiS_2_ bandgap range. Notably, each system is geometry optimized, converting experimentally determined atomic configurations into absolute DFT-determined ground states. Geometry optimization is especially necessary when changing orbital structures within the lattice, as atomic repositioning will occur as a result of evolving ground-state electron densities.

**Figure 1:**
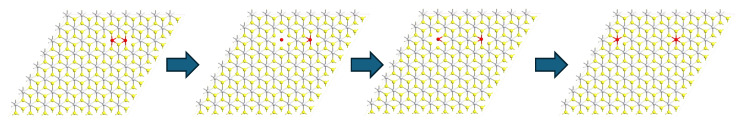
(**Left to right**): 1T-TiS_2_ supercells demonstrating transition-metal dopants directly substituted adjacent to each other and then with one, two, and three Ti atom locations mediating the exchange interaction along with surrounding S atoms. Here, grey atoms are Ti, yellow atoms are S, and the red atoms are the transition-metal atoms.

Magnetism induced by transition metal doping in TiS_2_ is analyzed by directly substituting titanium atoms within an 8 × 8 × 1 supercell with two transition metal atoms (V, Cr, Mn), as demonstrated in Figure 1. As the dopants provide additional electrons to the lattice, it is expected that they will gain a significant magnetic moment. Comparing the expected magnetic moment ($\mu_{B}$) with the calculated magnetic moment provides a benchmarking method to ensure that the calculations are properly evaluated. It should be noted that the SCAN function typically provides an overestimation for the magnetic moment in an attempt to correct for underestimations of the GGA calculation [44,46,47].

To examine the exchange interactions between TM dopants, we examine the energy differences between ferromagnetic and antiferromagnetic configurations. For each system (shown in Figure 1), two sets of initial spin parameters are tested. One set of initial spins corresponds with a ferromagnetic configuration, while the other corresponds with an antiferromagnetic alignment. Through a comparison of the total energies, we can determine the global ground state between the configurations. With the ground state known, we can evaluate the energy of the other configuration without any optimization, allowing us to compare only the electron shifts in the system. A comparison of the energies provides the exchange interaction.

Using a Heisenberg exchange analysis, the given energy requirement of a spin-flip for a transition-metal dopant corresponds with the interaction energy. The Heisenberg Hamiltonian is given by

(1) $$\hat{H}=-\sum_{i\neq j}J_{ij}{\vec{S}}_{i}\cdot {\vec{S}}_{j},$$

where $\hat{H}$ is the Hamiltonian operator, $J_{ij}$ is the exchange interaction term, and ${\vec{S}}_{i}$ and ${\vec{S}}_{j}$ are the spin operators for sites *i* and *j*, respectively. A positive exchange term $J_{ij}$ indicates a ferromagnetic system where spins energetically favor an aligned configuration, whereas a negative $J_{ij}$ corresponds with an antiferromagnetic configuration. To calculate the exchange interaction, we determine the total energy from both the geometry-optimized AFM and FM configurations. Once the ground state is determined, the non-ground state configuration is recalculated using the ground state structure without a geometry optimization.

In addition, determining the exchange interaction $J_{ij}$ as a function of the dopant separation helps provide the type of magnetic exchange present. A standard Heisenberg exchange $J_{ex}$ is controlled through orbital interactions and has either ferromagnetic or antiferromagnetic character. Additionally, the exchange typically drops off as $J_{ex}\propto e^{-r}$ as the magnetic centers are separated [48].

Another interaction is the Ruderman–Kittel–Kasuya–Yosida (RKKY) interaction $J_{RKKY}$ [49,50,51]. This interaction is typically governed by conduction electrons and provides modulation between ferromagnetic and antiferromagnetic character, dropping off in strength as the dopants are separated. Therefore, it is typically given by

(2) $$J_{RKKY}\propto \frac{1}{r^{3}}cos\left(2k_{F}r\right)$$

where *r* is the separation distance, and $k_{F}$ is the wavevector at the Fermi level. As such, examining the relationship between magnetic exchange energy and separation between magnetic atoms provides insight regarding the interaction mechanisms.

## 3. Results

Using density functional theory, we investigate the electronic and magnetic properties of the TM dopants in TiS_2_ by examining the electronic band structure, density of states, magnetic moment (as determined by the Mulliken population), and total energy of the systems. Additionally, a novel method is employed to determine the magnetic exchange energy between dopant atoms, which is established by approximating the magnetic exchange energy between two magnetic atoms as the energy required to flip the spin state of one of the atoms. Assuming titanium and sulfur atoms within a pristine TiS_2_ sample are non-magnetic due to the symmetry present in covalent bonding, we are capable of directly substituting transition metals (V, Cr, Mn) into titanium sites, resulting in an isolation of magnetism within the sample.

As V, Cr, and Mn provide additional electrons relative to the Ti atom they are replacing, it follows that each doped system should have a magnetic moment with respect to the transition metal dopants. For example, an 8 × 8 × 1 supercell of TiS_2_ with two titanium atom sites that have been directly substituted with vanadium atoms will have a magnetic moment of approximately one Bohr magneton at each dopant atom. DFT-based magnetic moment analysis provides a method for benchmarking our results against what we expect through fundamental chemistry (Table 1).

Within this paper, transition metal-doped systems are defined based on their geometries as shown in Figure 1. Prefixes 1 and 2 denote the number of transition metal dopants present within the lattice (1 or 2), and subscripts denote the titanium separations between dopants (1, 2, 3, or 4). In terms of vanadium dopant analysis, we consider that directly substituting a V atom for a Ti results in one additional electron in an otherwise perfectly covalently bonded system. The additional electron then provides a magnetic moment of one Bohr magneton, corresponding with a spin-$\frac{1}{2}$ system. The DFT calculation for the single-V-doped 1T-TiS_2_ system demonstrates a magnetic moment of 1.073 $\mu_{B}$ with a moment of 1.091 $\mu_{B}$ localized on the V atom, in agreement with expectations.

**Figure 2:**
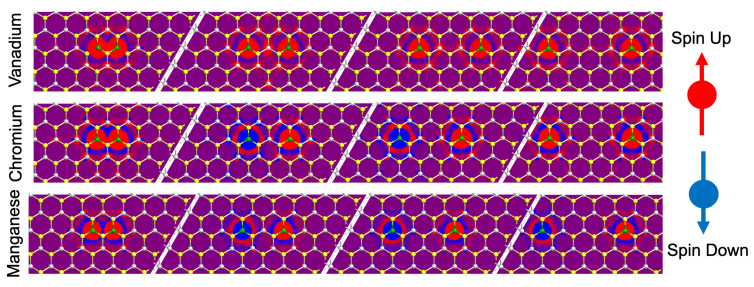
The electron density difference for each separation of the vanadium, chromium, and manganese systems. The red-colored density indicates spin up, and the blue indicates spin down.

**Figure 3:**
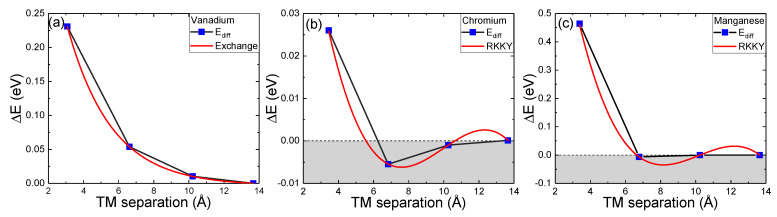
Energy difference (eV) as a function of transition metal (TM) separation (Å) for (**a**) vanadium, (**b**) chromium, and (**c**) manganese. The energy difference is the change in energy between respective antiferromagnetic and ferromagnetic configurations of the transition-metal dopants. This is consistent with the electron density difference calculations shown in Figure 2.

The systems with two V dopants all have total magnetic moments beyond two Bohr magnetons, which also agrees with expectations. As shown in Figure 2, each magnetic moment is positive, indicating ferromagnetic alignment for each dopant. The magnetic exchange interactions are analyzed through the total energy difference between the antiferromagnetic and ferromagnetic configurations. System 2V_1_ has the strongest magnetic exchange with 0.23 eV, which quickly decays as shown in Figure 3a. Systems 2V_2_ and 2V_3_ have magnetic exchanges of 0.0538 eV at 6.619 Å and 0.0103 eV at 10.218 Å respectively, with system 2V_4_ having 0 exchange at 13.667 Å. The V-doped system exhibits a standard Heisenberg exchange interaction, as indicated by its exponential decay behavior.

While the initial exchange interaction is a bit high, this is due to the initial separation of the TM atoms being fairly close together, which leads to considerable orbital overlap and a high exchange interaction. In most chalcogenide materials, the exchange is measured to be about an order of magnitude lower [52,53]. However, those materials have exchange distances closer to our second or third positions.

**Figure 4:**
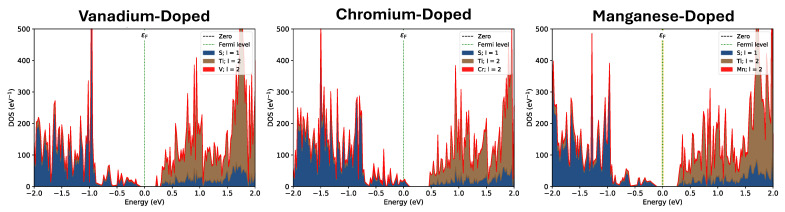
Density of states plots for each transition metal-doped system. V- and Mn-doped systems exhibit semiconducting behavior with Fermi levels located within the bandgap, whereas the Cr-doped system has states available at the Fermi level. In the legend, l is the orbital angular momentum, where 1 corresponds to *p*-orbitals and 2 corresponds to *d*-orbitals. The green dotted line indicates the Fermi level.

**Figure 5:**
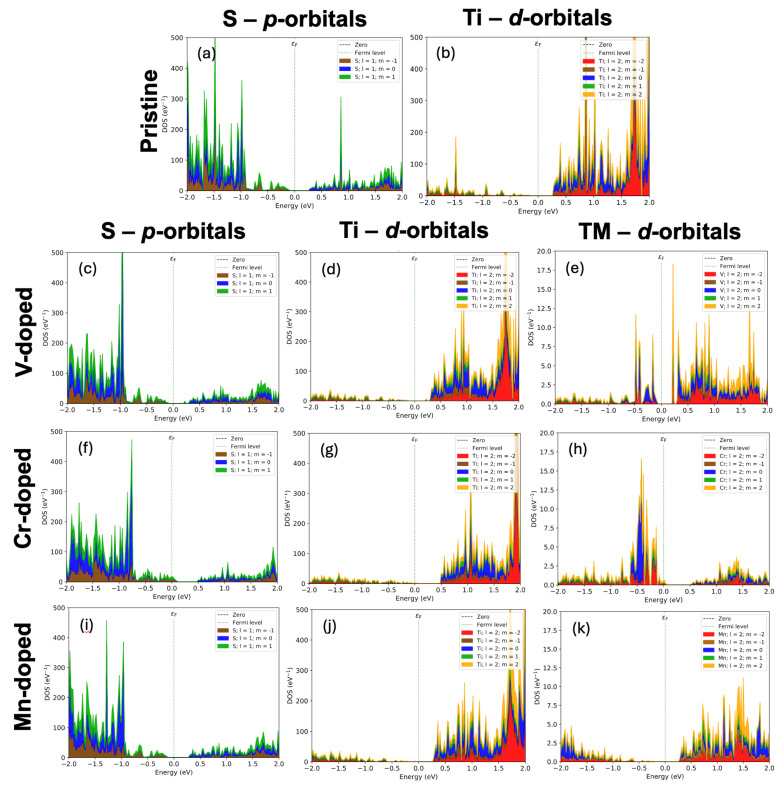
The projected density of states for the pristine TiS_2_ for the S-*p*-orbitals (**a**) and Ti-*d*-orbitals (**b**), as well as the S-*p*-orbitals, Ti-*d*-orbitals, and TM-*d*-orbitals for the double V- (**c**–**e**), Cr- (**f**–**h**), Mn- (**i**–**k**) doped systems, respectively. In the legend, l is the orbital angular momentum, where 1 corresponds to *p*-orbitals and 2 corresponds to *d*-orbitals. The m is the z-component of orbital angular momentum corresponding to the individual *d*-orbitals. The green dotted lines denote the Fermi level.

Additionally, the density of states for the V-doped system (shown in Figure 4) indicates that the system remains semiconducting upon the addition of the V atoms. Figure 5 breaks down the total density of states into the projected density of states per orbital, which indicates a Fermi level shift from S-*p* to Ti-*d* excitations (Figure 5a,b) as observed in the pristine TiS_2_ system to V-*d* to Ti-*d* (Figure 5c–e). It should be noted that in the legends of Figure 4 and Figure 5, l is the orbital angular momentum, where 1 corresponds to *p*-orbitals and 2 corresponds to *d*-orbitals. The m is the z component of orbital angular momentum corresponding to the individual *d*-orbitals. Overall, this suggests that the system interacts through orbital interactions, with the primary exchange being V to Ti.

In the Cr-doped system, Cr has two more electrons than Ti, and as such, we expect the Cr-doped systems to provide a spin-1 for each direct substitution within the TiS_2_ lattice. We first note that the single Cr system has a net magnetic moment of 2.914 $\mu_{B}$, which is high for a spin-1 system. However, referring to the density of states present in Figure 4, we note the presence of electronic states at the Fermi level, indicating conduction electrons within the system. In addition, the calculation of the magnetic moment is given by

(3) $$\mu_{S}=\sqrt{n(n+2)}\mu_{B},$$

where *n* is the number of unpaired electrons and provides further insight regarding the large magnetic moment. This spin-only formula is especially useful when analyzing transition metal complexes within the first series, as orbital momentum contributions are small [54,55,56,57,58]. Using the spin-only formula, the expected $\mu_{S}\approx 2.83\;\mu_{B}$ is much closer to our calculated value of 2.914 $\mu_{B}$.

Furthermore, the 2-Cr systems have a magnetic ground state configuration that depends on the separation of magnetic atoms present. At a separation of 3.453 Å, the system exists in a ferromagnetic ground state. For further separations, the spins prefer to be misaligned in an antiferromagnetic configuration (shown in Figure 3b). The oscillatory exchange interaction is a characteristic of the RKKY interaction, suggesting that conduction electrons play a role in the exchange of magnetism within the system.

Figure 4 shows the density of states for the Cr-doped system and clearly indicates that the system is weakly metallic by having an apparent density of states at the Fermi energy. Figure 5f–h shows that the states that appear at the Fermi level are a mixing of Cr-*d* and S-*p* orbitals, indicative of a conduction pathway. Overall, this further strengthens the argument that Cr is producing a distinct RKKY-like interaction.

The examination of the DOS suggests that the metallic characteristics for the Cr system are produced by a shift of the oxidation state from 4+ to 3+, which explains the higher calculated magnetic moment. The gain of an electron to the chromium atom would leave a hole in the lattice and allow for conductive states at the Fermi level.

Moving to the Mn-doped system, Mn has three more electrons than Ti, and we expect a spin-$\frac{3}{2}$ system. The single Mn-doped system has an overall net magnetic moment of 2.995 $\mu_{B}$ with the magnetic moment local to the Mn-dopant being 3.102 $\mu_{B}$, which agrees with our initial assumption of a spin-$\frac{3}{2}$ system. Similar to the Cr-doped systems, Mn also demonstrates a slight modulation of the magnetic ground state that depends on the separation between the exchanging atoms (shown in Figure 3c). The relative exchange interaction between the first and second separations is drastically reduced compared to the Cr systems. However, it is slightly antiferromagnetic and within the energy resolution of the calculation, which suggests that an RKKY interaction could govern it. Regardless of the nature of the mechanism, the Mn-doped system exhibits a dramatic drop-off in magnetic exchange energy as the separation increases.

To gain further insight, we re-examine the density of states. As shown in Figure 4, there are no conduction electrons at the Fermi level, and a clear bandgap exists, which does not support the idea of an RKKY-like interaction. Furthermore, looking at the projected density of states for the Mn-doped system (Figure 5i–k) reveals that the Mn-*d* orbitals are not greatly in play, which is likely why the exchange interaction drops off so dramatically. As such, further investigation is required before a dominant exchange interaction mechanism can be formally suggested for this system.

## 4. Conclusions

Magnetic exchange pathways are computationally investigated through transition metal doping within a monolayer TiS_2_ lattice. Given the non-magnetic response within a pristine sample, directly substituting various transition metals (V, Cr, and Mn) into Ti locations within the sample allows for a controlled method of isolating magnetic atoms and subsequent analysis. Four different systems are calculated for each dopant, each with a distinct atomic separation. Magnetic exchange interactions for each system are computed by setting two calculations, one with initial spin parameters corresponding to a ferromagnetic alignment and one with an antiferromagnetic alignment. The alignment that results in a lower total energy is then used and tested with the opposite alignment, without geometry optimization, to isolate any geometric factors contributing to the total energy difference.

V-substituted monolayer TiS_2_ is shown to demonstrate a standard Heisenberg exchange, as the relationship between the atomic separation of magnetic atoms and the magnetic exchange energy is a characteristic decaying exponential. The magnetic moment of V dopants is calculated to be 1 $\mu_{B}$, corresponding with the expected value. Cr-substitutions result in an oscillating magnetic exchange based on the separation of dopants, meaning that the magnetic alignment of Cr atoms alternates between ferromagnetic and antiferromagnetic as a function of separation. While this is characteristic of an RKKY interaction, there are no states available within the conduction band, requiring a more in-depth investigation to understand the underlying mechanisms at work. Cr dopants have a magnetic moment of 2.9 $\mu_{B}$, which is slightly higher than the expected value but agrees with spin-only considerations. Mn dopants technically result in an oscillating magnetic exchange interaction. However, the antiferromagnetic alignments are very small in energy, at less than 0.1 eV. In addition, there are no available conduction electrons at the Fermi level. Mn dopants are predicted to have a magnetic moment of 3.1 $\mu_{B}$, in agreement with the expected three electrons per dopant. Analysis of the density of states helps confirm these exchange mechanisms.

Overall, the presence of magnetic dopants in the Ti sites of TiS_2_ does produce magnetism in the system. Due to the weakly semiconducting nature of TiS_2_, the addition of extra electrons into the system appears to be able to create either a standard exchange or an RKKY exchange mechanism, depending on the presence of metallicity in the system. As such, careful consideration is required when analyzing magnetic exchange interactions within the TiS_2_ lattice, highlighting the potential for further investigation into robust magnetic coupling in these systems. As of the writing of this manuscript, there are no experimental realizations of these substitutions. However, this study does provide insight into how magnetic exchange couples through the lattice and at what distances this occurs, which can have implications on the magnetism caused by substitutional dopants, intercalated dopants, vacancies, or defects.

## Figures and Tables

**Table 1:** Magnetic analysis for 8 × 8 × 1 monolayer 1T-TiS_2_ systems with transition-metal dopants directly substituted into two Ti sites. Column 1 corresponds with Figure 1 and indicates which doped system is being considered. Column 2 provides the distance between dopants. Column 3 represents the difference in ground-state total energies for antiferromagnetic and ferromagnetic systems. Columns 4 and 5 indicate the magnetic moment corresponding to the dopant atoms. Column 6 provides the total magnetic moment for each system.

System	Dopant Separation (Å)	E_*A*_-E_*F*_ (eV)	$M_{{\mathrm{TM}}_{1}}$ ($\mu_{B}$)	$M_{{\mathrm{TM}}_{2}}$ ($\mu_{B}$)	M_*Total*_ ($\mu_{B}$)
1V	–	–	1.091	–	1.073
2V_1_	3.079	0.23096	1.231	1.149	2.077
2V_2_	6.619	0.05378	1.095	1.122	2.122
2V_3_	10.218	0.0103	1.082	1.095	2.086
2V_4_	13.667	0	1.144	1.075	2.179
1Cr	–	–	2.972	–	2.914
2Cr_1_	3.453	0.0260	2.939	2.94	5.502
2Cr_2_	6.829	−0.0055	2.943	−2.945	−0.013
2Cr_3_	10.258	−0.00099	2.948	−2.962	0
2Cr_4_	13.65	0.00012	−2.953	2.954	5.645
1Mn	–	–	3.102	–	2.995
2Mn_1_	3.388	0.46438	3.136	3.139	5.989
2Mn_2_	6.779	−0.0066	−3.115	3.104	−0.002
2Mn_3_	10.250	−0.00028	−3.120	3.109	−0.003
2Mn_4_	13.654	−0.00032	−3.119	3.0108	−0.002

## Data Availability

All data is available upon reasonable request to the authors.
